# Determination of the Effects of Duodenal Infusion Soy Protein Hydrolysate on Hepatic Glucose and Lipid Metabolism in Pigs Through Multi-Omics Analysis

**DOI:** 10.3389/fnut.2022.838617

**Published:** 2022-04-26

**Authors:** Zhongxin Li, Liren Ding, Weiyun Zhu, Suqin Hang

**Affiliations:** ^1^National Center for International Research on Animal Gut Nutrition, Jiangsu Key Laboratory of Gastrointestinal Nutrition and Animal Health, Laboratory of Gastrointestinal Microbiology, Nanjing Agricultural University, Nanjing, China; ^2^National Experimental Teaching Center for Animal Science, College of Animal Science and Technology, Nanjing Agricultural University, Nanjing, China

**Keywords:** soy protein hydrolysate, high plant protein, metabolome, transcriptome, liver, pigs

## Abstract

High animal protein intake increases hepatic lipid deposition and the risk of diabetes. However, the effects of high plant protein (HPP) intake on glycaemic responses and hepatic lipid metabolism in healthy people, as well as the underlying mechanisms, remain unclear. The current study explored the metabolomic and transcriptomic responses in the livers of pigs to assess the effects of HPP intake on host glucose and lipid metabolism. Sixteen pigs were infused with sterile saline or soy protein hydrolysate (SPH; 70 g/day) through a duodenal fistula twice daily during a 15 days experimental period. Hepatic metabolomic and transcriptomic analyses were performed, and the serum and hepatic biochemical parameters were measured. The results revealed that SPH infusion decreased serum glucose, hepatic triglyceride (TG), total cholesterol and low-density lipoprotein cholesterol levels, while it increased serum urea and eight hepatic amino acid levels (*P* < 0.05). Hepatic metabolomics displayed that SPH treatment produced seven different metabolites, four of which were related to lipid metabolism and one was related to glucose metabolism. In particular, lower (*P* < 0.05) glycocholic acid and glucose 1-phosphate levels and higher (*P* < 0.05) phosphatidylethanolamine (PE), arachidonic acid, prostaglandin F2α, l-carnitine and indole-3 acetic acid levels were observed following SPH infusion. A further metabolic pathway enrichment analysis found that these differential metabolites were mainly enriched in pathways related to lipid and glucose metabolism. Hepatic transcriptomics also demonstrated that multiple genes related to glucose and lipid metabolism were affected by SPH (*P* < 0.05). Together, SPH infusion reduced the hepatic TG levels by accelerating fatty acid β-oxidation and inhibiting TG synthesis. In addition, SPH infusion reduced the serum glucose levels by promoting hepatic glucose uptake and glycolysis. This study's result demonstrated that HPP intake regulated glycaemic responses and hepatic lipid metabolism in pigs without increasing the risk of hepatic lipid deposition and hyperglycaemia.

## Introduction

The intake of high-protein (HP) diet as a daily dietary practice or as part of a lifestyle pattern has recently been increasing. HP diet is related to weight loss and induces several positive metabolic effects, such as control and correction of certain risk factors related to the metabolic syndrome (1). However, there is scientific controversy about the safety of HP diet consumption. Some studies have reported that high animal protein intake increases hepatic lipid deposition and the risk of diabetes in healthy animals (2, 3). Compared to plant protein, high animal protein intake is often accompanied by the intake of animal fat and cholesterol. Several comparison experiments have proved that plant protein has a more prominent metabolic protection effect than animal protein (4, 5). However, the effect of high plant protein (HPP) intake on glycaemic responses and hepatic lipid metabolism in healthy animals, as well as its underlying mechanisms, remain unclear.

Soy protein is a high-quality protein derived from soybeans and provides an abundant source of dietary protein for humans and animals. For a long time, the associated health benefits of soy protein have received considerable attention, especially in regulating the host metabolism. In this study, soy protein was selected and hydrolysed into soy protein hydrolysate (SPH), which mimics the dietary proteins digested in the luminal chyme. The small intestine is a key site for dietary protein digestion and metabolites to be absorbed and transported into the liver. In this study, a duodenal fistula technique, which allows nutrients to directly act on the small intestine, was used. A pig was chosen as the model animal because its physiological structure is very similar to that of humans (6). Previous studies have investigated the effects of free amino acids and milk on hepatic amino acid absorption and metabolism using duodenal fistula pigs (7, 8).

Liver is a key organ for lipid metabolism. Hepatic lipid levels can be affected by various potential pathways, including lipid uptake and export, fatty acid β-oxidation, and lipid synthesis (9). Therefore, the mechanisms through which nutrients regulate hepatic lipid metabolism are complicated. Transcriptome analysis using RNA-sequencing (RNA-seq) can provide an unbiased overview of alterations that can occur at the molecular level (10). Metabolomics can analyse numerous metabolites from biological samples and provide an overview of metabolic changes related to dietary intake (11). Combined transcriptomic and metabolomic analyses in liver are highly valuable for comprehending the mechanisms through which HPP intake regulates the host lipid metabolism. In addition, serum glucose levels are closely related to hepatic glucose metabolism, and hepatic omics analysis facilitates the illustration of the mechanism of HPP intake for regulating glycaemic responses.

In this study, SPH was infused in the duodenum to evaluate the effects of HPP intake on host glucose and lipid metabolism and reveal the underlying mechanisms by which HPP diet regulates the serum glucose and hepatic lipid levels in a fistula pig model, through transcriptome and metabolome analyses.

## Materials and Methods

### SPH Preparation

The SPH was prepared following a previous method with slight modification (12). Briefly, the soy protein isolate (SPI, protein content 90%; Yuanye Biotechnology, Shanghai, China) was suspended in distilled water (10%, w/v) and adjusted to pH 2.0 using 2 N HCl. Then, the porcine pepsin (800–1000 U/mg protein; Yuanye Biotechnology) was added to hydrolyse SPI at an enzyme-to-substrate ratio of 1:100 (w/w). After 1 h of constant agitation at 37°C, the reaction was terminated by adjusting the solution pH to 7.0 with 2 N NaOH. The obtained hydrolysate was frozen at −20°C and lyophilised in a FreeZone 4.5 l Freeze Dry System (Labconco Co., Kansas City, MO, USA) for further use.

### Animals and Experimental Procedures

The Animal Welfare and Health Committee of Nanjing Agricultural University approved the experimental design and procedures. Sixteen castrated pigs (Duroc × Landrace × Large White, aged 50 days) with an initial weight of 14.5 ± 0.2 kg were obtained from a local commercial pig farm in Nanjing, China. All pigs were housed in individual metabolic cages under a controlled temperature of 25 ± 2°C and given unlimited access to water and feed. After a week of acclimatization, the pigs were made to fast for 12 h before installing a simple T-cannula (8.2 cm length, 10 cm width and 1.5 cm internal diameter) in the duodenum (just posterior to the pancreatic and bile duct) (13). After the surgery, all pigs were hypodermically injected with ceftriaxone sodium and treated with iodine tincture in the wound and adjacent skin for 1 week (twice a day) to avoid potential infection. After they fully recovered from the duodenal fistula surgery over a 2-week recuperation period, a 2-week short-term experiment of SPH on the secretion of an intestinal satiety hormone was performed (14). After a week of recovery, all pigs were randomly allocated to the control (CON, *n* = 8) group and SPH group (*n* = 8) with no differences in body weight (35.2 ± 0.3 kg) and feed intake. The entire experimental period was 15 days, during which the pigs in the CON and SPH groups were infused with 10 ml sterile saline and 10 ml SPH solution (70 g/day), respectively, through a duodenal fistula at 8:00 a.m. and 5:00 p.m. each day. The SPH solution was adjusted to pH 5.0, which is close to the native pH of porcine duodenum (15). The basal diet in the experiment was designed based on the National Research Council (NRC; 2012; Supplementary Table S1). The feed consumption of each pig was recorded every day to calculate average feed intake. In addition, the body weights of all pigs were recorded on days 1 and 16 to determine average weight gain.

### Sample Collection

All pigs were slaughtered after they fasted for 12 h on day 16. Blood samples (10 ml) were obtained from their jugular vein and centrifuged for 15 min (4°C, 3000 × g) to collect serum, which was then stored at −20°C. The livers of all pigs were obtained and immediately frozen in liquid nitrogen. The serum and liver tissues were thawed on ice before use.

### Biochemical Indicator Analysis

The serum biochemical indicators were determined using an Olympus AU2700 biochemical analyser (Olympus Optical Co., Ltd. Tokyo, Japan), including aspartate aminotransferase (AST), alanine aminotransferase, total protein, albumin, globulin, albumin/globulin (A/G), urea, glucose, total cholesterol (T-CHO), triglyceride (TG), high-density lipoprotein cholesterol (HDL-C) and low-density lipoprotein cholesterol (LDL-C). Hepatic TG (Cat No. A110-1-1), T-CHO (Cat No. A111-1-1), HDL-C (Cat No. A112-1-1) and LDL-C (Cat No. A113-1-1) levels were analyzed using commercial biochemical assay kits following the manufacturer's instructions (Nanjing Jiancheng Bioengineering Institution, Nanjing, China).

### Amino Acid Analysis

First, 100 mg of the liver samples were put in a 2 ml sterile centrifuge tube containing 0.5 ml of 0.5 mm silica/zirconium beads (BioSpec, Cat No. 11079105z) and 500 μl 0.9% saline solution. Homogenization was performed on FastPrep®-24 Instrument (MP Biochemicals, LLC, CA, USA) using 4–5 cycles at 6 m/s for 30–40 s with 2 min incubation time on ice between cycles. Then, the mixture was centrifuged (3000 × g, 4°C, 15 min) to obtain supernatants. Next, a 200 μl liver supernatant was collected and mixed with 1 ml 5% sulfosalicylic acid solution, and then maintained at 4°C for 30 min. Subsequently, the mixture was centrifuged for 20 min (4°C, 20,000 × g) and filtered with a filter (0.22 μm) for further analysis. Hepatic amino acid concentrations were measured using an LA8080 automatic amino acid analyser (Hitachi, Tokyo, Japan) following the method proposed by Kim et al. (16).

### Hepatic Metabolomic Profiles

Based on a previously described method (17), liver tissues were prepared from the CON and SPH groups for liquid chromatograph–mass spectrometer (LC–MS) analysis. The tissue samples (~50 mg) were homogenized in pre-cooled 50% methanol buffer (120 μl) using a high-throughput TissueLyser (Xinzhi Biotechnology Co., Ltd. Ningbo, China). The mixture was maintained on ice for 10 min and then centrifuged (4000 × g, 4°C, 20 min) to collect supernatants for subsequent analysis. All chromatographic separations were analyzed using an ultra-performance liquid chromatography (UPLC) system (Ultimate 3000; Dionex, CA, USA) equipped with an ACQUITY UPLC HSS T3 column (100 mm × 2.1 mm, 1.7 μm, Waters, MA, USA). The SIMCA-P software (version 13.0; Umetrics AB, Umea, Sweden) was used to establish the partial least-squares discriminant analysis (PLS-DA) model. The metabolites based on variable importance in the projection (VIP) value > 1.0 and *P*-value < 0.05 were considered as the differential metabolites between the CON and SPH groups. The metabolic pathways and metabolite set enrichment analysis were conducted using the MetaboAnalyst (version 5.0) tool.

### Transcriptome Sequencing Analyses

Total RNAs were isolated from the liver tissues using a TRIzol Reagent (Invitrogen, CA, USA, Cat No. 15596018) following the manufacturer's procedure. Then, Bioanalyzer 2100 and RNA 6000 Nano Lab Chip Kit (Agilent, CA, USA, Cat No. 5067-1511) were used to verify the RNA quantity and purity with RNA integrity number >7.0. High-quality RNA samples were collected for subsequent analysis. Under high-temperature conditions, divalent cations were used to fragment the purified mRNA into small fragments. The mRNA fragments were reverse transcribed to establish the cDNA library following the protocol for the mRNA-seq sample preparation kit (Illumina, San Diego, CA, USA, Cat No. RS-122-2101). Then, paired-end sequencing was performed on the Illumina HiSeq 4000 platform (LC Sciences, Santiago, CA, USA) at Shanghai Biozeron Biotechnology Co., Ltd. (Shanghai, China). To screen differential expression genes (DEGs) between the CON and SPH groups, the expression level for each gene was quantified following the fragments per kilobase of exon per million mapped reads method. We selected differential genes based on fold changes (FCs) ≥1.5 and *P*-values < 0.05. We used the ggplot2 package to draw a visual volcano map between different objects with the R software. GOATOOLS (https://github.com/tanghaibao/Goatools) and Kobas (http://kobas.cbi.pku.edu.cn/home.do) were used for gene ontology (GO) enrichment analysis and Kyoto Encyclopedia of Genes and Genomes (KEGG) pathway analysis, respectively.

### Quantitative Real-Time Polymerase Chain Reaction

Total RNA of liver tissues was extracted from the CON and SPH groups with the RNApure Total RNA Kit (Vazyme, Nanjing, China, Cat No. RC112-01). The concentration of the extracted RNA was determined using a Nano-Drop spectrophotometer (ThermoFisher Scientific, Wilmington, DC, USA) and adjusted to 500 ng/μl. The Roche SYBR Green PCR Kit (Vazyme, Nanjing, China, Cat No. Q221-01) was used to perform the polymerase chain reaction (PCR) in a final volume of 20 μl. Quantitative real-time PCR (qRT-PCR) was performed using the primers purchased from Invitrogen Life Technologies (Invitrogen, Shanghai, China), whose sequences are presented in Supplementary Table S2. For subsequent analysis, the average threshold cycle (C_t_) was calculated according to the triplicate qRT-PCR values of each cDNA. The formula 2^−Δ*ΔCt*^ was used to analyse the relative expression of the gene.

### Statistical Analysis

Biochemical indices, amino acid concentrations and qRT-PCR data were presented as mean ± standard error of the mean (SEM). All data were compared in SPSS 20.0 (SPSS Inc., Chicago, IL, USA) and graphs were drawn using GraphPad Prism 8.0.2 (La Jolla, CA, USA). Data were analyzed using a Student's *t*-test, and significant differences were observed when *P* < 0.05.

## Results

### Growth Performance and Protein Levels

The average daily feed intake, average daily gain, and ratio of feed to gain were not markedly different between the CON and SPH pigs (*P* > 0.05; Supplementary Table S3). The protein levels of the CON group (18.0% protein) and the SPH group (21.4% protein) were calculated based on the dietary composition (Supplementary Table S1) and average daily feed intake.

### Serum and Hepatic Biochemical Parameters

Table 1 demonstrates that lower (*P* < 0.05) serum TG, glucose and A/G levels and higher (*P* < 0.05) serum urea levels were observed in the SPH group. The levels of other serum parameters did not differ between the CON and SPH groups (*P* > 0.05). The hepatic TG, T-CHO and LDL-C levels decreased following SPH infusion (*P* < 0.05), whereas the HDL-C levels remained unaffected (*P* > 0.05).

**Table 1:** Serum and hepatic parameters in the CON and SPH groups.

**Items**	**CON**	**SPH**	***P*** **-value**
**Serum parameters**
AST (U/l)	40.43 ± 1.53	45.38 ± 5.01	0.372
ALT (U/l)	61.14 ± 2.55	69.88 ± 3.83	0.089
TP (g/l)	67.94 ± 2.20	69.75 ± 3.08	0.243
ALB (g/l)	37.37 ± 1.16	36.68 ± 1.05	0.288
GLOB (g/l)	30.57 ± 1.90	33.07 ± 2.16	0.084
A/G	1.27 ± 0.05	1.10 ± 0.04	0.027
Urea (mmol/l)	5.04 ± 0.29	6.47 ± 0.56	0.045
Glucose (mmol/l)	7.19 ± 0.37	6.06 ± 0.29	0.034
T-CHO (mmol/l)	2.38 ± 0.13	2.23 ± 0.16	0.789
TG (mmol/l)	0.55 ± 0.03	0.45 ± 0.02	0.018
HDL-C (mmol/l)	1.15 ± 0.07	1.11 ± 0.07	0.644
LDL-C (mmol/l)	1.39 ± 0.05	1.39 ± 0.11	0.953
**Hepatic parameters**
TG (mmol/gprot)	0.15 ± 0.02	0.11 ± 0.01	0.047
T-CHO (mmol/gprot)	0.04 ± 0.01	0.01 ± 0.00	0.026
LDL-C (mmol/gprot)	2.08 ± 0.30	1.19 ± 0.21	0.030
HDL-C (mmol/gprot)	9.60 ± 1.65	10.12 ± 1.25	0.808

*Values are mean ± SEM (n = 8). P < 0.05 was considered statistically significant. AST, aspartate transaminase; ALT, alanine aminotransferase; TP, total protein; ALB, albumin; GLOB, globulin; ALP, alkaline phosphatase; A/G, albumin/globulin; T-CHO, total cholesterol; TG, triglyceride; HDL-C, high-density lipoprotein cholesterol; LDL-C, low-density lipoprotein cholesterol*.

### Hepatic Amino Acid Concentrations

The concentration of amino acids in the liver, including valine, isoleucine, tyrosine, serine, lysine, aspartic acid, methionine and proline, were higher in the SPH group than the CON group (*P* < 0.05), whereas other amino acid concentrations did not show the difference between the two groups (*P* > 0.05; Table 2).

**Table 2:** Hepatic amino acid levels in the CON and SPH groups.

**Items**	**CON**	**SPH**	***P*** **-value**
Leucine	2.80 ± 0.07	3.24 ± 0.38	0.361
Valine	2.07 ± 0.08	2.77 ± 0.17	0.007
Isoleucine	1.14 ± 0.04	1.47 ± 0.09	0.013
Tryptophan	1.32 ± 0.05	1.55 ± 0.06	0.027
Tyrosine	1.56 ± 0.05	1.87 ± 0.08	0.011
Serine	2.34 ± 0.65	4.50 ± 0.55	0.027
Lysine	2.53 ± 0.11	3.24 ± 0.18	0.010
Aspartic acid	0.61 ± 0.16	1.05 ± 0.11	0.049
Glutamate	7.62 ± 0.13	8.47 ± 0.97	0.486
Methionine	0.80 ± 0.03	1.09 ± 0.07	0.005
Proline	2.84 ± 0.09	3.76 ± 0.24	0.011
Alanine	6.56 ± 0.23	6.88 ± 0.72	0.718
Cysteine	0.22 ± 0.00	0.22 ± 0.01	0.957
Phenylalanine	1.60 ± 0.03	1.89 ± 0.13	0.078
Histidine	1.22 ± 0.06	1.32 ± 0.05	0.189
Arginine	0.28 ± 0.04	0.44 ± 0.13	0.279
Glycine	9.89 ± 0.36	10.46 ± 1.09	0.680

*Values are mean ± SEM (n = 8); P < 0.05 was considered statistically significant*.

### Metabolome Profiles in Liver

**Figure 1:**
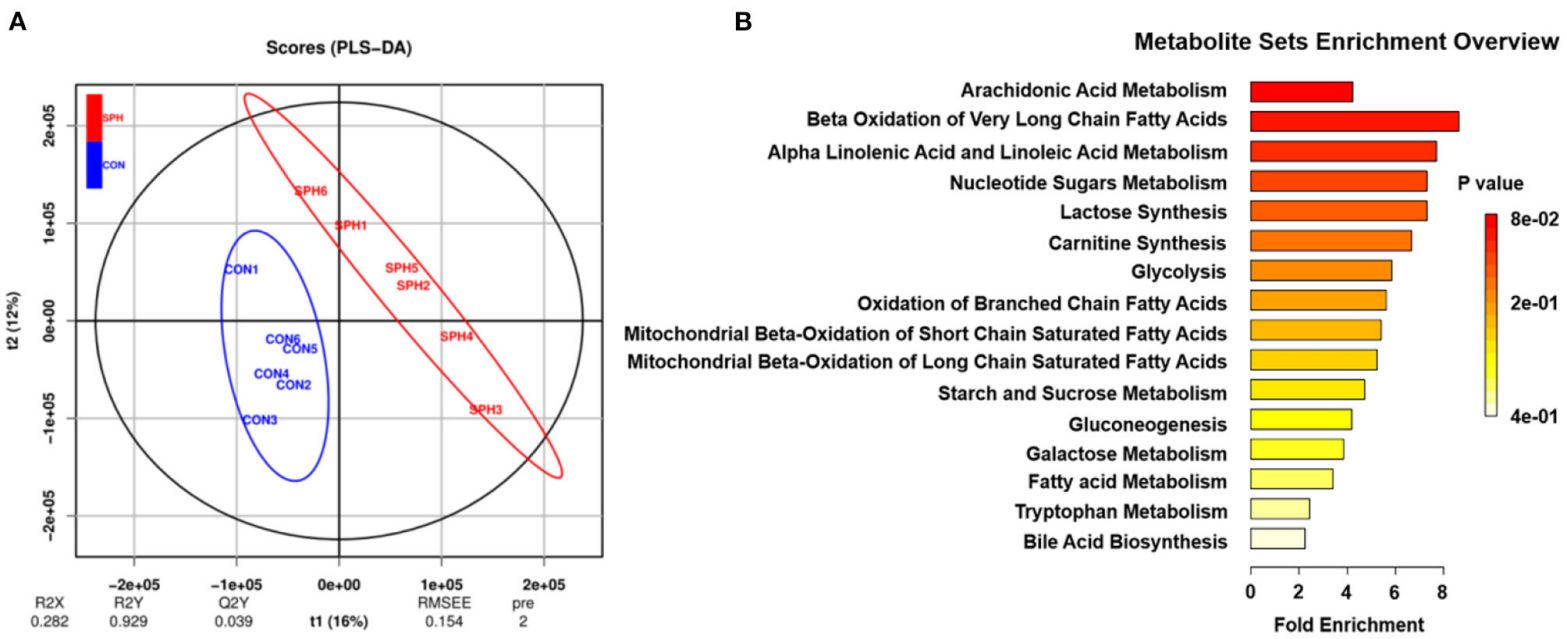
Liver metabolome of the CON and SPH groups. **(A)** PLS-DA of metabolites (n = 6). t1 explains 16% of the variation; t2 explains 12% of the variation. **(B)** Metabolite set enrichment overview map of the differential metabolites (VIP > 1.0 and *P* < 0.05) screened. The color of the rectangle indicates the *P*-value and its length indicates the pathway impact values. Longer length and darker colors indicate greater pathway enrichment and higher pathway impact values, respectively.

Metabonomic analysis was conducted using LC–MS to understand the hepatic metabolic changes that occurred after SPH treatment. The results of the PLS-DA analysis showed that samples in the SPH group were significantly discriminated from those in the CON group (Figure 1A). The differential metabolites between the two groups are presented in Table 3. SPH treatment significantly altered seven metabolites, of which four were involved in lipid metabolism, one was related to glucose metabolism and the other two were produced by amino acid metabolism. In particular, SPH infusion increased the levels of phosphatidylethanolamine (PE), arachidonic acid, prostaglandin F2α, l-carnitine and indole-3 acetic acid and decreased the levels of glycocholic acid and glucose 1-phosphate. The results of the metabolic pathway enrichment analysis revealed that these differential metabolites were mainly enriched in pathways related to lipid and glucose metabolism, including arachidonic acid metabolism, beta oxidation of very-long-chain fatty acids, alpha linolenic acid and linoleic acid metabolism, glycolysis and gluconeogenesis (Figure 1B).

**Table 3:** Differential metabolites in the liver between the CON and SPH groups.

**Metabolites**	**Metabolic subpathway**	**log_**2**_(FC)**	***P*-value**	**VIP**
l-carnitine	Carnitine synthesis	0.56	0.02	1.26
Glucose 1-phosphate	Glycolysis/gluconeogenesis	−0.75	0.02	1.77
Prostaglandin F2α	Arachidonic acid	0.54	0.03	1.10
	metabolism			
Phosphatidylethanolamine	Glycerophospholipid	0.94	0.03	1.73
	metabolism			
Arachidonic acid	Arachidonic acid	0.69	0.04	1.31
	metabolism			
Glycocholic acid	Bile acid biosynthesis	−0.91	0.04	2.56
Indole-3-acetic acid	Tryptophan metabolism	0.56	0.05	1.12

*log_2_(FC), the logarithmic transformation of fold change for the relative abundance of metabolites in SPH over CON. The P-value was obtained from the Wilcoxon–Mann–Whitney test and a value of < 0.05. VIP was obtained from the partial least-squares discriminant analysis model with a threshold of 1.0*.

### Hepatic Transcriptomics Analysis

**Figure 2:**
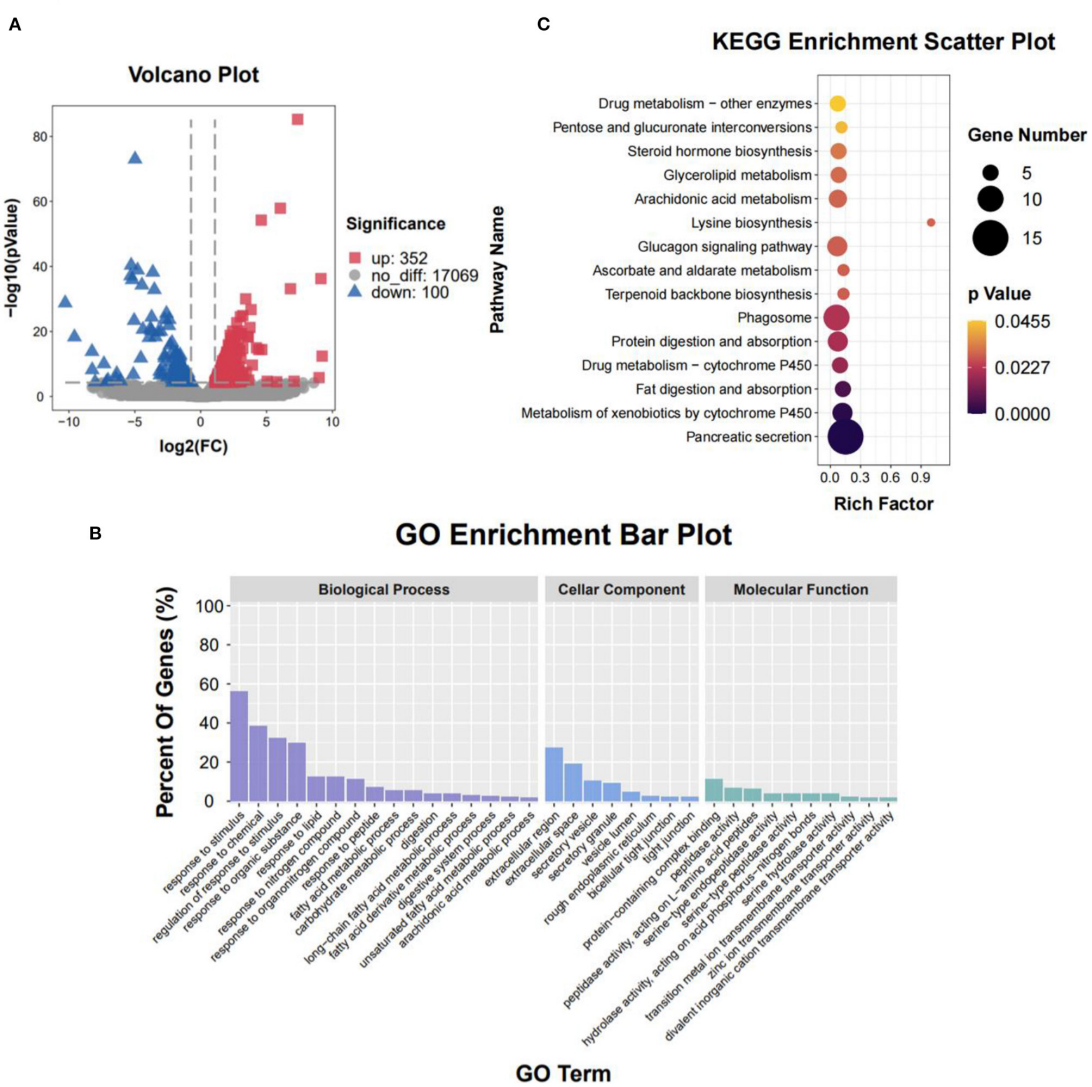
Liver transcriptome of the CON and SPH groups. **(A)** Volcano plot of distribution trends for differential expression genes (DEGs). Down-regulation and up-regulation are presented with blue and red dots, respectively (*n* = 3). **(B)** Gene ontology (GO) pathway enrichment analysis of DEGs. Each annotated sequence is divided into at least one GO term of the following: biological process, cellular component or molecular function. **(C)** Kyoto Encyclopedia of Genes and Genomes (KEGG) pathway enrichment analysis of DEGs. The size of the circles indicates the number of genes enriched in the pathway, and its color indicates the *P*-value.

To clarify the molecular processes of SPH infusion, RNA-seq was performed to analyse hepatic gene expression profiles. Following SPH treatment, 452 DEGs (352 up-regulated and 100 down-regulated) were identified from 17,521 genes (Figure 2A). The genes related to lipid metabolism, including elongation of very-long chain fatty acid protein 2 (*ELOVL2*), peroxisome proliferator-activated receptor alpha (*PPAR*α), long-chain acyl-CoA synthetase 6 (*ACSL6*), ethanolaminephosphotransferase 1 (*EPT1*), diacylglycerol O-acyltransferase 1 (*DGAT1*), diacylglycerol O-acyltransferase 2 (*DGAT2*), hydroxy-3-methylglutaryl-CoA synthase 1 (*HMGCS1*) and 3-hydroxy-3-methylglutaryl-CoA reductase (*HMGCR*), were up-regulated by SPH treatment. Amino acid metabolism-related genes, including amino acid transporters solute carrier family 38 member 1 (*SLC38A1*) and solute carrier family 7 member 1 (*SLC7A1*), were up-regulated in the SPH group. The glucose transport gene glucose transporter type 2 (*GLUT2*) and glycolysis genes, including 6-phosphofructo-2-kinase/fructose-2,6-biphosphatase 3 (*PFKFB3*), hexokinase domain component 1 (*HKDC1*) and lactate dehydrogenase B (*LDHB*), were up-regulated by SPH infusion (Table 4). Furthermore, GO enrichment analysis and KEGG pathway enrichment analysis were conducted on DEGs.

**Table 4:** Most relevant genes affected by SPH infusion in the liver.

**Metabolic pathway**	**Gene name**	**Gene symbol**	**log_**2**_(FC)**	***P*-value**
Carbohydrate metabolism	Glucose transporter type 2	*GLUT 2*	1.33	3.72E-07
	Hexokinase domain component 1	*HKDC1*	1.03	8.33E-04
	6-phosphofructo-2-kinase/fructose-2,6-bisphosphatase 3	*PFKFB3*	1.68	1.35E-11
	Lactate dehydrogenase B	*LDHB*	4.59	4.77E-55
	Fructose-1,6-bisphosphatase isozyme 2	*FBP2*	−0.90	2.22E-05
Amino acid metabolism	Amino acid transporters solute carrier family 38 member 1	*SLC38A1*	0.92	1.89E-03
	Solute carrier family 7 member 1	*SLC7A1*	0.95	1.62E-03
Lipid metabolism	Elongation of very-long chain fatty acid protein 2	*ELOVL2*	1.14	3.88E-05
	Peroxisome proliferator-activated receptor alpha	*PPARα*	2.32	8.33E-18
	Long-chain acyl-CoA synthetase 6	*ACSL6*	4.19	2.55E-03
	Hydroxy-3-methylglutaryl-CoA synthase 1	*HMGCS1*	0.99	3.38E-04
	3-hydroxy-3-methylglutaryl-CoA reductase	*HMGCR*	1.65	1.94E-10
	Ethanolaminephosphotransferase 1	*EPT1*	2.94	5.26E-20
	Diacylglycerol O-acyltransferase 1	*DGAT1*	−0.89	1.19E-05
	Diacylglycerol O-acyltransferase 2	*DGAT2*	−1.70	1.17E-05

*log_2_(FC), the logarithmic transformation of fold change for expression levels of genes in SPH over CON. The gene expressions are significantly affected when FC > 1.5 and P < 0.05*.

The result of the GO enrichment analysis is presented in Figure 2B. At the biological process level, several GO terms in the SPH group were involved in lipid metabolism, such as the response to lipid, fatty acid metabolic process, unsaturated fatty acid metabolic process and arachidonic acid metabolic process. Moreover, the GO term of the carbohydrate metabolic process was enriched in the SPH group. At the cellular component level, most genes were significantly enriched in the extracellular region and extracellular space. At the molecular function level, most genes were represented in the protein-containing complex binding and peptidase activity.

When the DEGs were portrayed to KEGG pathways, SPH treatment significantly changed 15 pathways (Figure 2C). Several pathways were related to lipid metabolism, including fat digestion and absorption, arachidonic acid metabolism, glycerolipid metabolism and steroid hormone biosynthesis. In addition, SPH treatment significantly affected lysine biosynthesis, glucagon signaling pathway and other pathways. Both GO and KEGG analyses significantly changed the pathways related to lipid and carbohydrate metabolism.

### Confirmation of Sequencing Results Obtained by qRT-PCR

**Figure 3:**
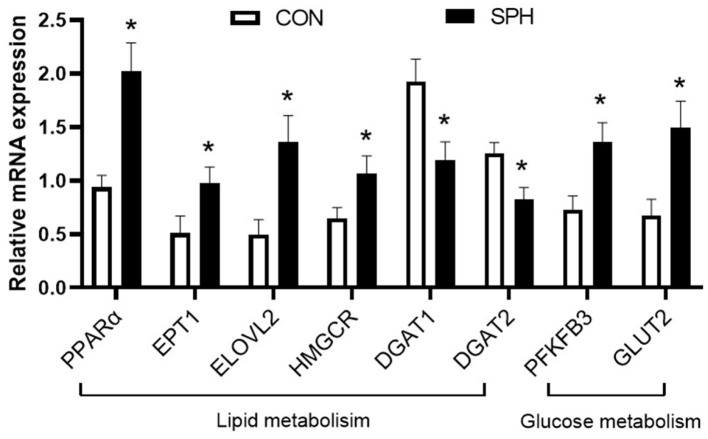
qRT-PCR analysis of DEGs in the CON and SPH groups. Values are mean ± SEM (*n* = 8). *P < 0.05.

Quantitative real-time PCR (qRT-PCR) analysis was performed, focusing on the most prominent pathways, to validate the transcriptome analysis result (Figure 3). As the SPH treatment strongly altered hepatic lipid metabolism, the following six lipid metabolism-related genes were verified: *PPAR*α, *EPT1, ELOVL2, HMGCR, DGAT1* and *DGAT2*. In addition, *PFKFB3* involved in glycolysis and *GLUT2* involved in glucose uptake were also determined. These genes were consistent with the expression pattern observed in the transcriptomic analysis, which confirmed the reliability of the transcriptome data.

## Discussion

Dietary protein is mainly digested and absorbed in the small intestine, and its metabolites can be transported to the liver through blood circulation for a direct effect on hepatic metabolism (18). In this study, SPH increased eight amino acid levels in the liver, including lysine, methionine and serine, and increased the expression of genes related to amino acid transport, including *SLC38A1* and *SLC7A1*, which are involved in the transport of neutral and cationic amino acids, respectively (19). The amino acids absorbed into the liver participate in multiple metabolic pathways, and excess amino acids produce urea through the urea cycle. In this study, SPH infusion also increased serum urea production. Previous studies indicated that rats and pigs fed an HP diet increased amino acid uptake and activated the hepatic urea cycle and increased serum urea production (2, 20).

Previous studies have demonstrated that HP diet decreases postprandial blood glucose levels and improves glucose tolerance in individuals with type 2 diabetes (21, 22). However, Sluijs et al. (3) revealed that high animal protein (meat, milk and casein) intake increases the risk of diabetes in healthy people, which is a metabolic disease characterized by hyperglycaemia. In this study, SPH infusion reduced the serum glucose levels, indicating that HPP intake does not promote the occurrence of hyperglycaemia. The metabolite profile showed that SPH infusion reduced the levels of hepatic glucose 1-phosphate, which suggested that SPH accelerated hepatic glucose metabolism. Transcriptome analysis showed that glycolysis-related genes *HKDC1, PFKFB3* and *LDHB* were up-regulated by SPH. Fructose-1,6-bisphosphatase can hydrolyse fructose-1,6-bisphosphate to produce fructose-6-phosphate (3). *HKDC1* encodes a protein that catalyzes glucose to produce glucose-6-phosphate (23). The key enzyme of glycolysis, 6-phosphofructo-1-kinase, is positively regulated by fructose 2,6-bisphosphate, which is catalyzed and synthesized by PFKFB3 (24). LDHB catalyzes the interconversion of lactate and pyruvate (25). Therefore, SPH infusion accelerated hepatic glycolysis. Stepien et al. (26) found that Wistar rats fed a diet containing 50% milk protein inhibited glycolysis in the liver after 14 days of intervention. The reasons for this difference are unclear, but it is attributable to the different protein types. In addition, *GLUT2* was up-regulated by SPH. Studies on *Glut2*-deficient mice have shown that *GLUT2* is necessary for glucose uptake but not for glucose output (27). The expression of *GLUT2* indicated that the circulation of glucose uptake was promoted in the liver, which might be in response to the acceleration of glycolysis.

Triglycerides are a significant indicator when assessing lipid metabolism. Garcia et al. (28) proved that compared to a low-protein diet, HP diet reduces hepatic TG deposition in steatosis mice. Diaz-Rua et al. (2) investigated the safety of long-term intake of HP diet and found that Wistar rats fed a high-casein diet had increased liver TG deposition and exhibited health risk signs after 4 months of intervention. In the current study, SPH infusion decreased hepatic TG levels, indicating that HPP intake did not increase the risk of TG deposition. Kozaczek et al. (4) performed RNA-seq on liver samples of obese Zucker rats fed with SPI for 16 weeks and found that SPI might have inhibited the liver steatosis by enhancing lipid conversion and transport. However, the mechanisms through which HPP intake inhibited hepatic TG accumulation might be different in normal and obese animal models. In the present study, both metabolome and transcriptome analyses showed that SPH treatment affected the lipid metabolism pathways. In addition, *PPAR*α was up-regulated following SPH treatment. Tovar et al. (29) showed that, in the liver of obese rats, soy protein supplementation promoted *PPAR*α expression, leading to increased β-oxidation of fatty acid. In addition, SPH increased hepatic *ACSL6* expression and l-carnitine levels in this study. ACSL6 converts long-chain fatty acids to acyl-CoAs (30). Previous studies showed that HP intake increased the concentration of lysine and methionine, which increased l-carnitine production in the liver in pigs (31, 32). l-carnitine can transport acyl-CoA into the mitochondrial matrix to promote fatty acid β-oxidation, which can be biosynthesized by l-lysine and l-methionine as substrates in the body (33), suggesting that SPH infusion accelerates fatty acid β-oxidation in the liver.

Lipid reduction might also be related to the inhibition of TG synthesis. French et al. (34) showed that HP diet reduced the expression of the fatty acid synthase, thereby inhibiting hepatic fat deposition in obese Zucker rats. In their study, *DGAT1* and *DGAT2* were down-regulated by SPH, which are the key enzymes that catalyze TG formation from diacylglycerol (DG) (35). This indicates that SPH treatment inhibited TG synthesis. Furthermore, SPH infusion increased PE production and up-regulated *EPT1* expression. *EPT1* was identified and believed to be responsible for catalyzing the final step of the PE synthesis (36). Previous studies also found that dietary soy protein supplementation altered the fatty acid profile and increased PE content in the liver in mice and pigs (37, 38). The synthesis of TG and PE requires a common substrate, DG. When the synthesis of either TG or PE was blocked, DG was redistributed to synthesize the other (35). Therefore, SPH infusion can break the balance of TG and PE syntheses by regulating the hepatic gene expression, thereby reducing TG deposition. SPH infusion increased hepatic arachidonic acid levels and up-regulated *ELOVL2* expression in this study. In mammals, *ELOVL2* encodes an elongase that catalyzes the elongation of monounsaturated and polyunsaturated fatty acids (39). Unsaturated fatty acids are an important component of PE, whose synthesis provides the necessary substrate for PE synthesis.

Additionally, the effect of HPP intake on cholesterol metabolism has been evaluated. Tovar et al. (29) confirmed that compared to casein intake, soy protein supplementation significantly reduced hepatic cholesterol levels in obese mice. In their study, SPH infusion reduced the hepatic T-CHO and LDL-C levels, indicating that HPP intake did not cause hepatic cholesterol accumulation (40). A previous study demonstrated that hepatic cholesterol homeostasis was affected by lipoprotein synthesis, secretion to the blood and reuptake into the liver (9). However, the serum T-CHO and LDL-C levels were not altered in this study. Hence, the reduction in the hepatic cholesterol levels by SPH infusion might not be due to alterations in the lipoprotein flow. Yang et al. (41) revealed that soy protein intake reduced hepatic cholesterol levels of non-alcoholic steatohepatitis rats, which resulted from an increase in the bile acid synthesis and secretion. However, the metabolome analysis showed that SPH reduced the hepatic glycocholic acid levels. Nagao et al. (42) found that SPI reduced the lipodystrophy-induced hepatic cholesterol accumulation by inhibiting the expression of cholesterol synthesis genes. Unexpectedly, *HMGCS1* and *HMGCR* were up-regulated by SPH infusion, which are the key enzymes that promote the *de novo* synthesis of cholesterol (43). This difference may be attributable to the different models used. Most previous studies have used lipid deposition models in animals to evaluate the effect of soy protein on lipid metabolism (44–46), whereas the present study used a normal pig model. Cholesterol is an indispensable macronutrient that has several significant functions in animals. Therefore, cholesterol homeostasis is vital for maintaining appropriate cellular and systemic functions. There might be a negative feedback mechanism that regulates the cholesterol synthesis. When SPH treatment reduces the cholesterol levels to the threshold, the liver initiates a negative feedback mechanism to accelerate cholesterol synthesis and inhibit cholesterol degradation.

Note that SPH infusion significantly affects the glucagon signaling pathway, which is related to glucose and lipid metabolism. Therefore, the glucagon signaling pathway might be involved in SPH-regulated hepatic metabolism; however, this needs to be further verified. These findings provide new data for evaluating the safety and hepatic metabolic effects of HPP intake in healthy animals.

## Conclusion

**Figure 4:**
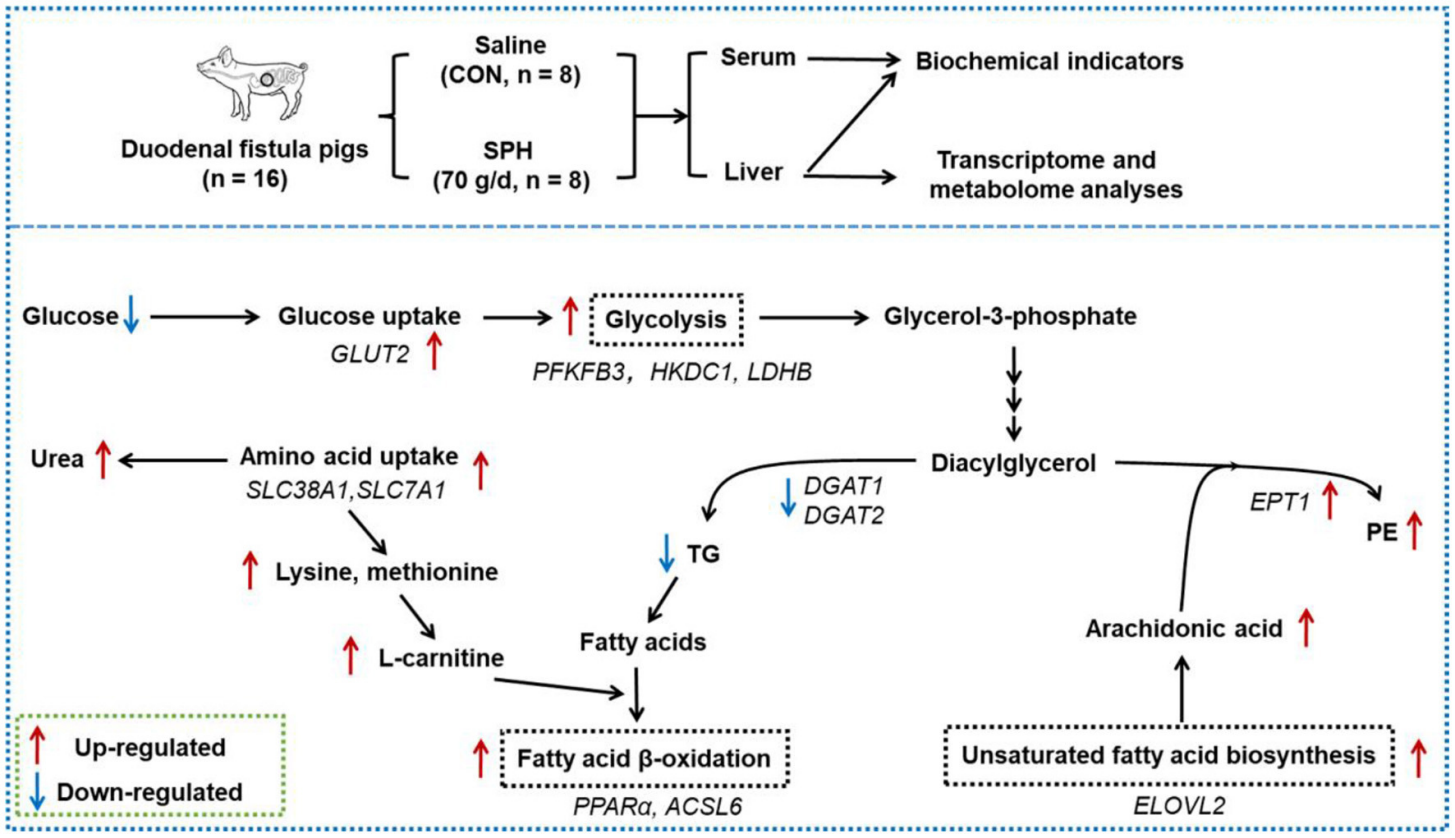
Effects of the duodenal infusion SPH on liver metabolism. The red arrows represent the up-regulated level of genes and metabolites in the SPH group compared to the control (CON) group; the blue arrows represent the down-regulated level of genes and metabolites in the SPH group compared to the CON group.

High plant protein intake regulated glycaemic responses and hepatic lipid metabolism, but did not increase the risk of hepatic lipid deposition and hyperglycaemia in pigs. SPH infusion accelerated fatty acid β-oxidation and inhibited TG synthesis, thereby reducing the hepatic TG levels. The inhibition of TG synthesis by soy protein might be related to the promotion of PE synthesis. SPH infusion reduced the serum glucose levels by promoting hepatic glucose uptake and glycolysis (Figure 4). SPH infusion inhibited cholesterol accumulation in the liver and initiated a negative feedback mechanism, which maintains cholesterol homeostasis. These findings validated the effects of HPP on host glucose and lipid metabolism in healthy animals.

## Data Availability Statement

The datasets presented in this study can be found in online repositories. The names of the repository/repositories and accession number(s) can be found in the article/Supplementary Material.

## Ethics Statement

The animal study was reviewed and approved by Experimental Animal Welfare Ethics Committee of Nanjing Agricultural University.

## Author Contributions

SH and ZL conceived and designed the experiments. ZL and LD performed the experiments. ZL and SH analyzed the data and drafted the manuscript. SH and WZ provided the funding. All authors read and approved the final manuscript.

## Funding

This research was funded by the National Key Basic Research Program of China (2013CB127301).

## Conflict of Interest

The authors declare that the research was conducted in the absence of any commercial or financial relationships that could be construed as a potential conflict of interest.

## Publisher's Note

All claims expressed in this article are solely those of the authors and do not necessarily represent those of their affiliated organizations, or those of the publisher, the editors and the reviewers. Any product that may be evaluated in this article, or claim that may be made by its manufacturer, is not guaranteed or endorsed by the publisher.
