# Supplementary material for: Determination of the Effects of Duodenal Infusion Soy Protein Hydrolysate on Hepatic Glucose and Lipid Metabolism in Pigs Through Multi-Omics Analysis
Source: Front Nutr. 2022 Apr 26;9:838617. doi: 10.3389/fnut.2022.838617 (PMC9087715; doi:10.3389/fnut.2022.838617)
Supplement: Supplementary file 1 [file Data_Sheet_1.DOCX]

***Supplementary Material***

Supplementary Tables

Table S1 Dietary composition and nutritional component (air dry basis).

| **Items** | **Composition, %** |
| --- | --- |
| Ingredients |  |
| Corn | 59.81 |
| Soya bean meal | 25.80 |
| Wheat flour | 5.00 |
| Rice bran meal | 3.00 |
| Corn germ meal | 3.00 |
| L-Lysine, 98.5% | 0.11 |
| DL-Methionine, 99.0% | 0.04 |
| L-Threonine, 99.0% | 0.04 |
| L-Tryptophan, 99.0% | 0.01 |
| Calcium hydrogen phosphate | 0.40 |
| Limestone | 1.00 |
| NaCl | 0.50 |
| Acidifying agent | 0.40 |
| Zinc oxide | 0.20 |
| Choline chloride | 0.10 |
| Mould inhibitor | 0.05 |
| Phytase | 0.02 |
| Compound enzyme | 0.02 |
| Mineral premix^a^ | 0.50 |
| Nutrition level, % |  |
| NE, MJ/Kg | 10.19 |
| Crude protein | 18.00 |
| Crude fat | 2.63 |
| Crude fiber | 3.79 |
| Crude ash | 5.03 |
| Lysine | 1.10 |
| Methionine | 0.34 |
| Tryptophan | 0.21 |
| Threonine | 0.75 |
| Valine | 0.87 |

^a^ Premix supplied the following per kg complete diet: vitamin A, 3,800 IU; vitamin D_3_, 800 IU; vitamin E, 9 mg; vitamin B_1_, 1 mg; vitamin K_3_, 1 mg; vitamin B_2_, 2 mg; vitamin B_6_, 1.2 mg; vitamin B_12_, 10 µg; nicotinic acid, 10 mg; biotin, 50 µg; folic acid, 0.4 mg; iron (as FeSO_4_ · H_2_O), 80 mg; zinc, 80 mg; iodine (as KI), 0.14 mg; Se (as Na_2_SeO_3_), 0.25 mg; copper as (CuSO_4_ · 5H_2_O), 5 mg; and Mn (as MnSO_4_ · H_2_O), 3 mg.

Table S2 Description of primers used in the RT-qPCR analysis of gene expression.

| **Gene** | **Forward** | **Reverse** |
| --- | --- | --- |
| *HMGCR* | CTACATTGCCTGTGGTCAGGATG | CCGATCTCTATGGATGGCATGGT |
| *PPARα* | AGACCGCAGATCTCAAGTCTCTC | ATGACGAAAGGCGGGTTATTGC |
| *PFKFB3* | GACCCGCTACCTCAACTGG | TGGCATCAAAAACCGCAAT |
| *ELOVL2* | ATTCTTCACCACCAGCGAGG | TGCCTGGCTGTTATCACTCG |
| *GLUT2* | AAGTCGAGGCCTATGATCTGACTAA | GGAAGAGGCATATCAGGACTCTACT |
| *EPT1* | GATGGTGTGGATGGAAAGCAA | GCCATGGTCAAAGAGTTCTCCTA |
| *DGAT1* | AGGACGGACACGGAT | GAGCAGTCAGCAAA |
| *DGAT2* | TCCTGTCTTTCCTCGTGC | ACCTTTCTTGGGGCGTGT |
| *β-actin* | CCACGAAACTACCTTCAACTC | TGATCTCCTTCTGCATCCTGT |

*HMGCR*, 3-hydroxy-3-methylglutaryl-coenzyme A reductase; *PPARα*, peroxisome proliferator-activated receptor alpha; *PFKFB3*, 6-phosphofructo-2-kinase/fructose-2,6-bisphosphatase 3 isoform X2; *ELOVL2*, elongation of very long chain fatty acids protein 2 isoform X1; *GLUT2*, glucose transporter type 2; *EPT1*, ethanolaminephosphotransferase 1; *DGAT1*, diacylglycerol o-acyltransferase 1; *DGAT2*, diacylglycerol-O-acyltransferase 2.

Table S3 Growth performance in the CON and SPH groups.

| **Items** | **CON** | **SPH** | ***P-*value** |
| --- | --- | --- | --- |
| Average daily feed intake (kg/d) | 1.73 ± 0.07 | 1.61 ± 0.07 | 0.236 |
| Average daily gain (kg) | 0.87 ± 0.04 | 0.75 ± 0.06 | 0.095 |
| Feed conversion ratio | 2.06 ± 0.10 | 2.13 ± 0.18 | 0.737 |

Values are mean ± SEM (n = 8). *P* < 0.05 implies statistically significant.
